# Disruption in normal correlational patterns of metabolic networks in the limbic circuit during transient global amnesia

**DOI:** 10.1093/braincomms/fcad082

**Published:** 2023-03-21

**Authors:** Shailendra Segobin, Cyrielle Renault, Fausto Viader, Francis Eustache, Anne Lise Pitel, Peggy Quinette

**Affiliations:** Normandie University, UNICAEN, PSL Research University, EPHE, INSERM, U1077, CHU de Caen, Cyceron, Neuropsychologie et Imagerie de la Mémoire Humaine, 14032, Caen, Normandie, France; Normandie University, UNICAEN, PSL Research University, EPHE, INSERM, U1077, CHU de Caen, Cyceron, Neuropsychologie et Imagerie de la Mémoire Humaine, 14032, Caen, Normandie, France; Normandie University, UNICAEN, PSL Research University, EPHE, INSERM, U1077, CHU de Caen, Cyceron, Neuropsychologie et Imagerie de la Mémoire Humaine, 14032, Caen, Normandie, France; Normandie University, UNICAEN, PSL Research University, EPHE, INSERM, U1077, CHU de Caen, Cyceron, Neuropsychologie et Imagerie de la Mémoire Humaine, 14032, Caen, Normandie, France; Normandie University, UNICAEN, PSL Research University, EPHE, INSERM, U1077, CHU de Caen, Cyceron, Neuropsychologie et Imagerie de la Mémoire Humaine, 14032, Caen, Normandie, France; Normandie University, UNICAEN, INSERM, U1237, PhIND ‘Physiopathology and Imaging of Neurological Disorders’, Cyceron, 14032, Caen, Normandie, France; Normandie University, UNICAEN, PSL Research University, EPHE, INSERM, U1077, CHU de Caen, Cyceron, Neuropsychologie et Imagerie de la Mémoire Humaine, 14032, Caen, Normandie, France

**Keywords:** PET, transient global amnesia, metabolism, memory, emotion

## Abstract

Transient global amnesia is characterized by the sudden apparition of severe episodic amnesia, mainly anterograde, associated with emotional changes. Even though the symptoms are stereotyped, cerebral mechanism underlying transient global amnesia remains unexplained and previous studies using positron emission tomography do not show any clear results or consensus on cerebral regions impacted during transient global amnesia. This study included a group of 10 transient global amnesic patients who underwent ^18^F-fluorodeoxyglucose positron emission tomography during the acute or recovery phase of the episode and 10 paired healthy controls. Episodic memory was evaluated with the encoding-storage-retrieval paradigm and a story recall test of the Wechsler’s memory scale and anxiety was assessed with the Spielberger scale. We used statistical parametric mapping to identify modifications of whole-brain metabolism. Regarding hypometabolism, there was no brain region systematically affected in all transient global amnesic patients and the comparison between amnesic patients and controls did not show any significant differences. To better understand the specific implication of the limbic circuit in the pathophysiology of transient global amnesia, we then conducted a correlational analysis that included regions of this network. Our findings showed that in healthy controls, regions of the limbic circuit seem to operate in a synchronized way with all regions being highly correlated to each other. On the opposite, in transient global amnesic patients, we observed a clear disruption of this normal correlational patterns between regions with the medial temporal lobe (the hippocampus, parahippocampal gyrus and amygdala) included in one cluster and the orbitofrontal cortex, anterior and posterior cingulate gyrus and thalamus gathered in the other one. Given the individual variability in the time course of transient global amnesia, the direct comparison between a group of patients and controls does not seem to favour the identification of subtle and transient alterations in regional metabolism. The involvement of an extended network, such as the limbic circuit, seems more likely to explain the symptoms of patients. Indeed, the synchronization of regions within the limbic circuit seems to be altered during transient global amnesia, which could explain the amnesia and anxiety observed in transient global amnesic patients. The present study thus deepens our understanding of the mechanisms underlying not only amnesia but also the emotional component of transient global amnesia by considering it as a disruption in the normal correlational patterns within the limbic circuit.

## Teaser

Segobin *et al*. examine brain metabolism using ^18^F-fluorodeoxyglucose PET in 10 patients with transient global amnesia in acute/recovery phases. They found a disruption of the correlational pattern observed between regions of the limbic circuit. Alteration of brain synchronization could explain both amnesia and anxiety observed in transient global amnesia.

## Graphical Abstract

**Graphical abstract:**
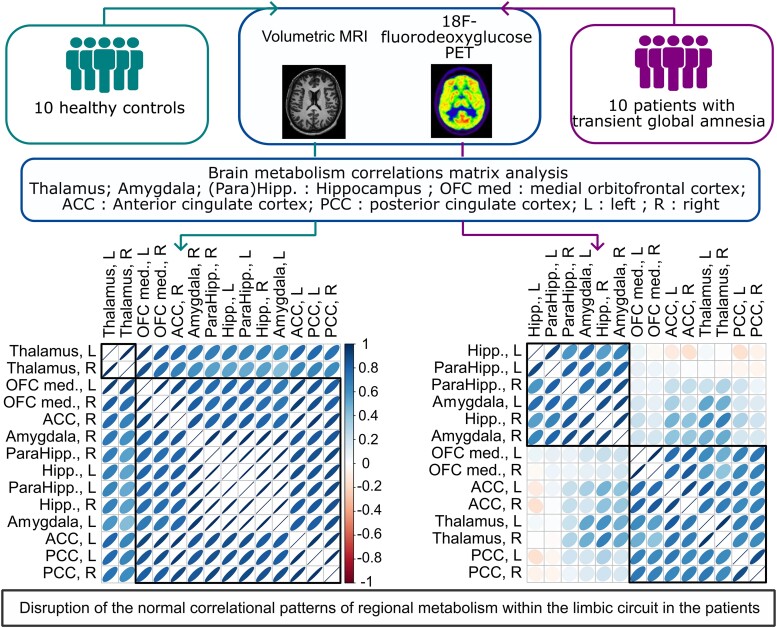

## Introduction

Even though transient global amnesia (TGA) has been clearly defined by Hodges and Warlow in 1990,^1^ its pathophysiology remains enigmatic. This stereotyped neurological syndrome is characterized by the sudden onset of transient and massive anterograde amnesia. It is associated, during the acute phase, with more variable retrograde amnesia, iterative questions and temporal disorientation,^2^ and is accompanied by mood changes including increased anxiety.^2^ The following recovery phase is characterized by the disappearance of temporal disorientation and iterative questions but with the persistence of deficient memory scores. According to Hodges and Warlow criteria, patients gradually recover within 24 hours and the attack does not result in any long-term neuropsychological sequels.^3^ One of the striking characteristics of TGA is the absence of volume deficits in anatomical magnetic resonance imaging (MRI) examinations.^4^

TGA is usually classified as a hippocampal amnesia since the severe episodic memory deficits observed in TGA patients are supposed to be related to hippocampus alterations. Indeed, hyperintensities on diffusion-weighted imaging (DWI) MRI are often detected in the hippocampus and more specifically in the CA1 field of the hippocampal cornu ammonis (CA) in TGA patients.^4-6^ Interestingly, the lesions are mainly observed 12–48 hours after the TGA onset. These abnormalities have been interpreted as a consequence of functional pathological brain events having occurred during the acute phase of TGA.^5^ The vulnerability of the temporal lobe was also highlighted in two single-case studies using fMRI, with a reduction in the Blood-Oxygen-Level-Dependent (BOLD) signal being observed while TGA patients were encoding new information during the acute phase.^7,8^

The use of positron emission tomography (PET) allows the study of brain functions at a molecular level. It is thus more sensitive to subtle and/or transient changes occurring with a pathological event and could be relevant to use in TGA. The PET scan requires the injection of a radiotracer, such as the [^18^F]-2-fluoro-2-deoxy-d-glucose (FDG-PET), and allows, in that case, a measure of the cerebral metabolic rate of glucose. The measure of glucose consumption, necessary for neurone operations, likely reflects localized synaptic activity.^9,10^ However, PET studies conducted in TGA patients are very scarce with only six single-case studies (Table 1) and only two using an FDG-PET examination. It is indeed extremely difficult to recruit a group of TGA patients, especially during the acute and recovery phases. Besides, the use of PET scan requires access to specific facilities and expertise, which makes the study of brain metabolism in TGA patients even more challenging. All previous PET investigations used a region of interest (ROI) method and showed heterogeneous results with altered metabolism (hyper- or hypometabolism) in various regions^11,12^ including the hippocampus,^13-15^ thalamus^11^ or amygdala.^14^

**Table 1:** Previous PET studies conducted in TGA patients during acute or recovery phases

Study	Population	TGA phase at the PET scan time (time from onset)	Imaging analysis	Main results
Volpe *et al*., 1983	*N* = 1	Acute (na)	PET (CMRO_2_) quantitative	Hypometabolism in the bilateral **hippocampus**
Heiss *et al*., 1992	*N* = 1	Recovery (+6 h)	PET (CMRglu) quantitative	Hypometabolism in bilateral **internal temporal lobe**
Baron *et al*., 1994	*N* = 1	Recovery (+5 h)	PET (CMRO_2_) quantitative (ROI)	Hypometabolism in the right **thalamus**, **right lenticular nucleus** and right **lateral frontal cortex**
Eustache *et al*., 1997	*N* = 1	Recovery (+11 h)	PET (CMRO_2_) quantitative (ROI)	Hypometabolism in the left **lateral temporal lobe**, left **lateral frontal** and left **lenticular nucleus**
Guillery *et al*., 2002	*N* = 1	Acute (+3h30)	PET (CMRO_2_) quantitative (SPM, ROI)	Tendency to a hypermetabolism in the left **posterior hippocampus**
	*N* = 1	Recovery (+7 h)	PET (CMRO_2_) quantitative (SPM, ROI)	Subtle but significant hypometabolism in the **amygdala** and left **posterior hippocampus**
Gonzalez-Martinez *et al*., 2010	*N* = 1	Acute (+2 h)	PET (CMRglu) quantitative	Hypometabolism in the left **hippocampus**

ROI: region of interest; CMRO_2_/glu: cerebral metabolic rates for oxygen/glucose. na: data not available.

Even though hippocampal dysfunction may contribute to TGA, there exists no consensus in the literature regarding the pathophysiological mechanisms that govern this pathology. In addition, the sole implication of the hippocampus cannot explain the emotional aspect of this syndrome. Indeed, in parallel to amnesia, TGA patients in the acute phase also present emotional changes with increased anxiety and more depressed mood.^2,16^ Those changes, noticed in papers on TGA,^17^ can be associated with the higher increase of the cortisol level observed in patients during TGA compared to time-matched day level^18^ or to a control group, before and after a stressful experimentation.^19^ These findings suggest that TGA patients might be hypersensitive to stress and have difficulties coping with it.^20^ However, the origin of those emotional symptoms and their relationships with amnesia are not clearly understood. They could either reflect a psychological reaction to the sudden onset of amnesia, or a symptom at the same level as amnesia due to brain dysfunction of regions involved in both memory and emotion.^21^

No region thus seems to be solely responsible for the symptoms seen in TGA. In fact, there is growing evidence towards TGA being rather due to a brain network dysfunction,^22-26^ explaining the absence of- or heterogeneity in- results when investigating the implication of regions independently of each other rather than them working together as a network. The observation of both emotional and amnesic symptoms points to the implication of the limbic system, a set of brain regions known to support both emotions and memory.^27^ In this perspective, a dysfunction in synaptic activity within the limbic circuit could explain the combination of severe memory disorders and emotional changes observed in TGA patients. Studies on the limbic system have highlighted the potential existence of two sub-networks, one being involved in emotion [including the amygdala, orbitofrontal cortex (OFC) and the anterior cingulate cortex (ACC)] and the other in episodic memory [including the hippocampus, parahippocampal gyrus, thalamus and posterior cingulate cortex (PCC)].^28,29^ Also in accordance with the literature regarding the regions involved in amnesia,^30,31^ it seems thus more pertinent to study brain alterations in TGA patients within these regions as a network rather than each as their own entity.

The goal of this study was to provide a better understanding of the pathophysiological mechanisms contributing to amnesia and anxiety in TGA. We aimed at explaining the absence of structural alterations and the heterogeneity in the previous PET results by testing the hypothesis that TGA is a transient network disorder^24^ affecting the limbic circuit and leading to both memory deficits and anxiety. The objective was thus to examine brain metabolism using FDG-PET in a group of TGA patients in acute or recovery phases. We first used a voxel-based method to conduct an individual analysis of the variability among the group of TGA patients and to describe the pattern of glucose metabolism in the whole brain for the entire group. Then, since the inherent heterogeneity in the clinical state of TGA patients seems to make group comparisons difficult to interpret on their own, we also used an ROI method and a correlational approach to specifically investigate brain metabolism in the regions of the limbic system.^28,29^ More generally, this study aims at providing novel insights into the understanding of human memory and its interactions with emotion using rare data collected in TGA patients.

## Materials and methods

### Participants

Ten TGA patients admitted to the emergency department of Caen University Hospital (CHU) (France) were recruited, over 5 years, by expert neurologists (Table 2) according to the Hodges and Warlow criteria.^1^ Neurological examination (except regarding memory impairment) was normal. Individual TGA contexts are detailed in Supplementary Data 1. Ten healthy control subjects matched for age and education were recruited. All participants gave their written informed consent to participate in this study, approved by the local ethic committee.

**Table 2:** Clinical and demographic characteristics of patients with TGA

	Age (years)	Gender	Education (years)	TGA duration^a^ (hours)	Precipitating events^b^	Risk factors
PA01	72	Woman	14	7	Emotional	None
PA02	59	Woman	11	8	None	None
PA03	65	Woman	12	5	Emotional	AHT, migraine
PA04	69	Woman	9	9	Physical	Anxiety
PA05	61	Woman	15	7	Physical	None
PA06	60	Woman	15	3.25	Physical	None
PA07	69	Woman	11	3	None	AHT, migraine
PA08	54	Woman	9	4.5	Emotional	na
PA09	67	Man	12	8	None	None
PA10	62	Man	17	6	None	None
Patients (mean ± sd)	63.8 ± 5.27	2 Men | 8 Women	12 ± 2.54	6.1 ± 2.1		
Controls (mean ± sd)	63.7 ± 4.7	1 Man | 9 Women	11.8 ± 2.71	Not applicable		

a The duration of TGA is estimated based on: the onset of the attack (easily identifiable by the suddenness of the amnesia and related by a witness) and the end of the acute phase that is sometimes difficult to pinpoint as the recovery is gradual. We therefore used several indicators to determine the end of the attack: the day of the episode we paid attention to the clinical signs as the temporal disorientation, the iterative questioning and the ability to memorize information (such the neuropsychologist’s name); and the day after we checked the duration of the TGA by studying the length of the lacunar amnesia (Quinette *et al*. 2006).

b Incidents occurring immediately before the attack of a few days before, which might have influenced the occurring of TGA (more details in Supplementary Data 1). They can be emotional (stressful event, dispute) or physical (training, temperature change).

na: data not available. AHT: arterial hypertension.

### Experimental design

**Figure 1:**
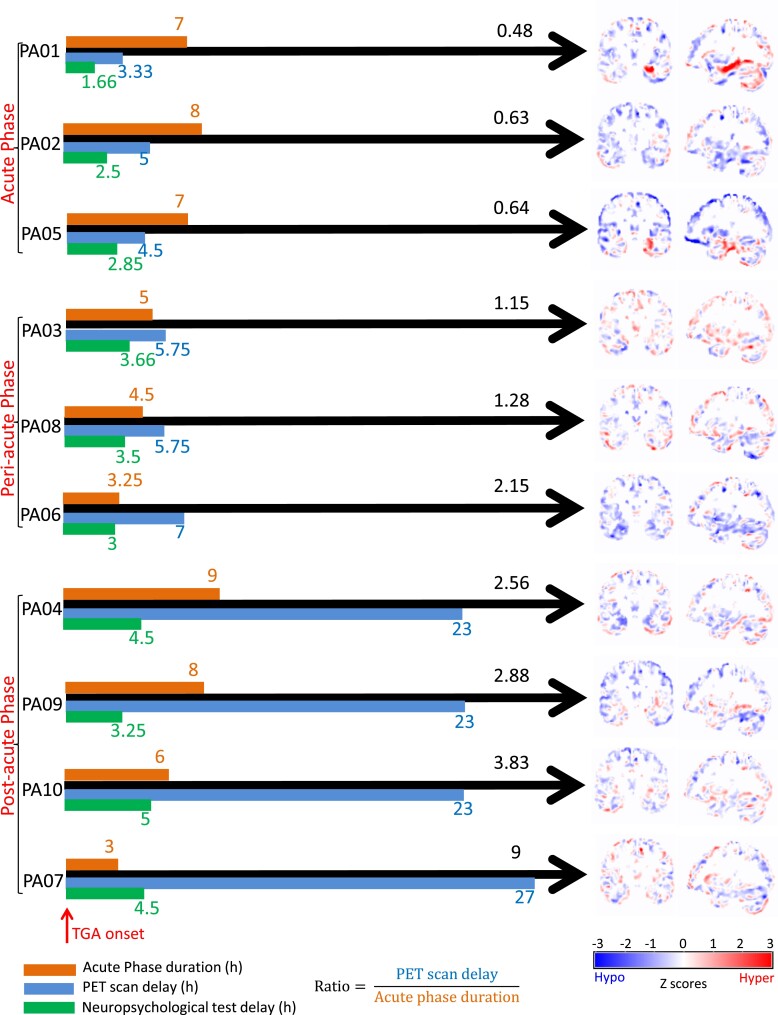
**Individual timelines of examinations in TGA patients.** The individual timelines show, for each patient, the duration of the acute TGA phase and the timing of the psychological/neuropsychological evaluations and PET examination. Patients are presented in ‘acute phase’ or ‘recovery phase’ based on the timing of the PET scan in regard to the onset of the episode. They are classified according to a ratio taking account the timing of the PET scan compared to the duration of the acute phase. A ratio <1 indicates that the PET scan was conducted during the acute phase.

At the emergency department, after the diagnosis of TGA, patients followed a specific research protocol (individual timelines are reported in Fig. 1). First, psychological and neuropsychological evaluations were performed in the emergency department as early as possible after the diagnosis. Second, patients underwent an FDG-PET scan at CHU in the department of Nuclear Medicine and Medical biophysics. Depending on the arrival time of the patient at the hospital, the delay to obtain an FDG dose and the availability of the scanner, the PET scan was conducted between 3h25 and 27 h after the onset of the TGA. Finally, an anatomical MRI examination was conducted days or weeks (3–107 days) after the TGA episode at the neuroimaging centre (Cyceron, Caen) depending on the slot availability. Healthy controls followed the same protocol, at the same place (CHU and Cyceron) and in the same conditions as patients.

### Psychological and neuropsychological examination

#### Anxiety

State and trait anxiety was assessed by parts A and B of the state-trait anxiety inventory (STAI).^32^ For this scale, the higher the score (out of 80), the more anxious the patient.

#### Memory

Episodic memory was evaluated with the encoding—storage—retrieval (ESR) task.^33^ In this test, the participant has to learn a list of 16 words. During the learning (encoding) phase, the first two words are presented to the participant who is asked to produce a sentence with each word to promote deep encoding. After the encoding of these first two words, an immediate cued recall task is conducted to ensure that the items have been correctly encoded. The procedure is then repeated with the next two words until the end of the list, at the end of this learning process, a free recall task is conducted. Then, a recognition task (with the correct word being presented among three distractors) is carried out.

Since the PET scan was not systematically conducted exactly at the same time as the psychological and neuropsychological assessment, a story recall test [story A of the logical memory subset of the Wechsler’s memory scale (WMS)^34^] was used just before or after the PET examination to estimate amnesia. The WMS test, based on spontaneous encoding, is a more sensitive test than the ESR task and therefore pertinent to use at the PET scan time.

### Neuroimaging examination

#### Data acquisition

##### MRI

Each participant underwent a volumetric high-resolution T1-weighted MRI. All MRI data were acquired at the neuroimaging centre (Cyceron, Caen), on a 3 Tesla Philips Achieva scanner (*Eindhoven*, Netherlands) (Fast Spin Echo, FSE; sagittal; repetition time = 20 ms; echo time = 4.6 ms; flip angle = 10°; 180 slices; slice thickness = 1 mm; field of view = 256 × 256 mm^2^; matrix = 256 × 256).

##### FDG-PET

Data were collected at CHU on the Siemens Biograph HD + PET device (*Siemens Healthcare*, Germany) with isotropic resolution of 2.1 mm^3^ (Field of View, FOV = 158 mm). 370 MBq of ^18^F-fluorodeoxyglucose (FDG) were injected intravenously 30 minutes before data acquisition in list mode.^35^ The images were reconstructed with ordered-subset expectation maximization—point spread function iterative reconstruction (3 iterations, 21 subsets, a voxel size of 1 × 1 × 2 mm^3^).

#### Data pre-processing

##### MRI data

The voxel-based morphometry (VBM) toolbox of SPM12 software (Statistical Parametric Mapping, Wellcome Trust Center for Neuroimaging, London, UK) was used to segment T1-weighted MRI data into grey matter, white matter and cerebrospinal fluid. The images were then spatially normalized into the Montreal Neurological Institute (MNI) standardized space (voxel size = 1.5 mm^3^; matrix = 121 × 145 × 121). Individual distortion parameters were saved to be applied to the PET images.

##### PET data

For each subject, PET scans were individually realigned onto their respective MRI images and normalized into the MNI space by reapplying the previous distortion parameters. The final voxel size was 79 × 95 × 79 with an isotropic resolution of 2 mm^3^. No correction for partial volume effects was performed retrospectively as partial volume correction had already been applied per se via reconstruction of PET data with resolution modelling.^36^

For the quantitative normalization, the cerebellum was used as a reference region in order to control for the inter-individual variations in PET measurements and avoid an artificial increase of metabolism values in altered regions.^37^ The cerebellum has already been used in a previous study^38^ of our laboratory.

### Statistical analyses

#### Psychological and neuropsychological profile

R 4.0.3 (R Core Team, 2020) and RStudio 1.4.1106 (RStudio Team, 2019) software were used for psychological and neuropsychological data analysis. Standard descriptive statistics were used, as well as nonparametric Mann–Whitney *U* tests to compare patients and controls (*stats* 4.0.3 package).

Individual Z-scores were also calculated based on the performance of the control subjects and were considered as impaired for a Z-score inferior at –1.85 or superior at +1.85 depending on the variables (adjusted threshold for a small sample size, degree of freedom equal to 8 for *N* = 10).

#### Whole-brain voxel-based between-group comparisons

Voxel-based analyses were performed using two-sample *t*-tests in SPM12 to compare glucose metabolism between patients and controls. Both contrasts (controls > patients and controls < patients) were carried out to examine hypometabolism and hypermetabolism, respectively. A threshold of *P* < 0.001 (uncorrected for multiple comparisons) and a cluster size of 25 voxels (corresponding to 200 mm^3^) have been applied. For voxel-based analyses, all post-processed PET data were smoothed with a Gaussian kernel of 10 mm^3^.

#### Z-score frequency maps

The variability of the individual patterns of brain abnormalities in patients was analyzed through z-score maps. Voxel-wise z-score maps were calculated for each patient using mean and standard deviation from the control group. Each z-score map was then thresholded at +1.85 in order to obtain a binary map of all voxels deemed hypermetabolic and at −1.85 for a binary map of hypometabolic voxels. The hypermetabolic and hypometabolic binary maps were then summed to obtain a hypermetabolic frequency map and a hypometabolic frequency map respectively. Both maps contained voxels ranging between 0 and 10 and would indicate the number of TGA patients having significant hypermetabolic or hypometabolic voxels within the cohort. For example, if a voxel in the hypometabolic frequency map contained a value equal to 4, it implied that 4 TGA patients had significant hypometabolism in that voxel. The number of hypo or hypermetabolic voxels necessary to get a cluster with a significant size was thresholded at a minimum of 25 voxels.

#### Correlation between brain metabolism and temporal aspect

To measure the relationships between regional brain metabolism and the earliness of the PET scan examination in TGA patients, voxel-based correlations were performed in SPM12 between glucose uptake and a ratio that considers both the time at which the PET scan was performed and the estimated total duration of the acute phase of TGA (ratio = PET scan delay in hours/estimated acute phase duration in hours).

#### Correlations matrix

To better understand the specific implication of the limbic circuit in the pathophysiology of TGA, we conducted a correlational analysis that included the hippocampus, parahippocampal gyrus, thalamus, posterior and ACC, amygdala and OFC^28,29^ (Table 3). The AAL atlas (automated anatomical labelling)^39^ was used to extract metabolic values from the specified regions. For patients and controls, regional metabolism measures were individually computed into a correlation matrix using R software (*corrplot* 0.84 package; Wei & Simko, 2017). In each group, a method of hierarchical clustering was applied to the correlation coefficients to classify the different regions into two clusters based on the magnitude and direction of their correlations.

**Table 3:** Regions of the limbic circuit integrated in the correlations matrix

Hippocampus (Hipp.)
Parahippocampal gyrus (ParaHipp.)
Amygdala
Thalamus
Anterior cingulate cortex (ACC)
Posterior cingulate cortex (PCC)
Medial orbitofrontal cortex (med OFC)

## Results

*Psychological and neuropsychological profile.* Anxiety and memory raw scores of the patients are reported in Table 4.

**Table 4:** Psychological and neuropsychological tests results

	Anxiety scores		Memory scores		
	STAI state/80 (z-score)	STAI trait/80 (z-score)	ESR immediate cued recall/16	ESR free recall/16 (z-score)	ESR recognition/16
PA01	69 **(4.68)**^a^	53 (1.5)	12	1 **(−9.22)**^a^	5
PA02	76 **(5.56)**^a^	53 (1.5)	9	1 **(−9.22)**^a^	8
PA03	51 **(2.43)**^a^	56 **(1.98)**^a^	16	5 **(−4.88)**^a^	12
PA04	38 (0.79)	46 (0.38)	15	1 **(−9.22)**^a^	12
PA05	64 **(4.06)**^a^	46 (0.38)	14	2 **(−8.13)**^a^	7
PA06	53 **(2.68)**^a^	34 (−1.55)	15	4 **(−5.97)**^a^	16
PA07	42 (1.30)	40 (−0.59)	16	9 (−0.54)	16
PA08	53 **(2.68)**^a^	51 (1.18)	12	4 **(−5.97)**^a^	16
PA09	56 **(3.05)**^a^	32 (−1.88)	12	3 **(−7.05)**^a^	9
PA10	46 (1.80)	47 (0.54)	16	9 (−0.54)	16
Patients mean score	54.8*	45.8	13.7*	3.9*	11.7*
Controls mean score	31.67	43.67	16	9,5	16

*Significant difference compared to controls or norms (Mann Whitney’s tests, *P* < 0.05).

a Z-scores were considered as pathological for a threshold inferior at –1.85 or superior at +1.85.

The ESR task was conducted as early as possible after the diagnosis. For the cued recall and recognition tests, z-scores calculation was not possible due to the absence of variability in the controls’ scores.

When considered as a group, patients were significantly more anxious than controls (*U* = 4.5, *P* = 0.001) on the STAI state but not on the trait scale. Z-scores analysis revealed that compared with controls, seven patients presented state anxiety and one had trait anxiety.

Regarding episodic memory performance, patients had lower results than controls on the cued recall (*U* = 85, *P* = 0.002), free recall (*U* = 93, *P* < 0.001) and recognition (*U* = 80, *P* = 0.006) tasks of the ESR. At the time of the PET analysis, patients still had episodic memory deficits on the WMS story recall (*U* = 76, *P* = 0.002) when considered as a group, with seven of them presenting altered individual z-scores (Table 4). All patients had at least one memory score (either ESR or WMS) significantly impaired, with very low z-scores.

As expected, we found in TGA patients a significant nonparametric correlation between the earliness of the neuropsychological assessment and the immediate cued recall (*ρ* = 0.76; *P* = 0.009), the free recall (*ρ* = 0.67; *P* = 0.03) and the recognition raw scores (*ρ* = 0.72; *P* = 0.01).

### Whole-brain voxel-based between-group comparisons

The comparison between TGA patients and controls did not show any significant differences at *P* < 0.001 (uncorrected for multiple comparisons).

### Z-score frequency maps

**Figure 2:**
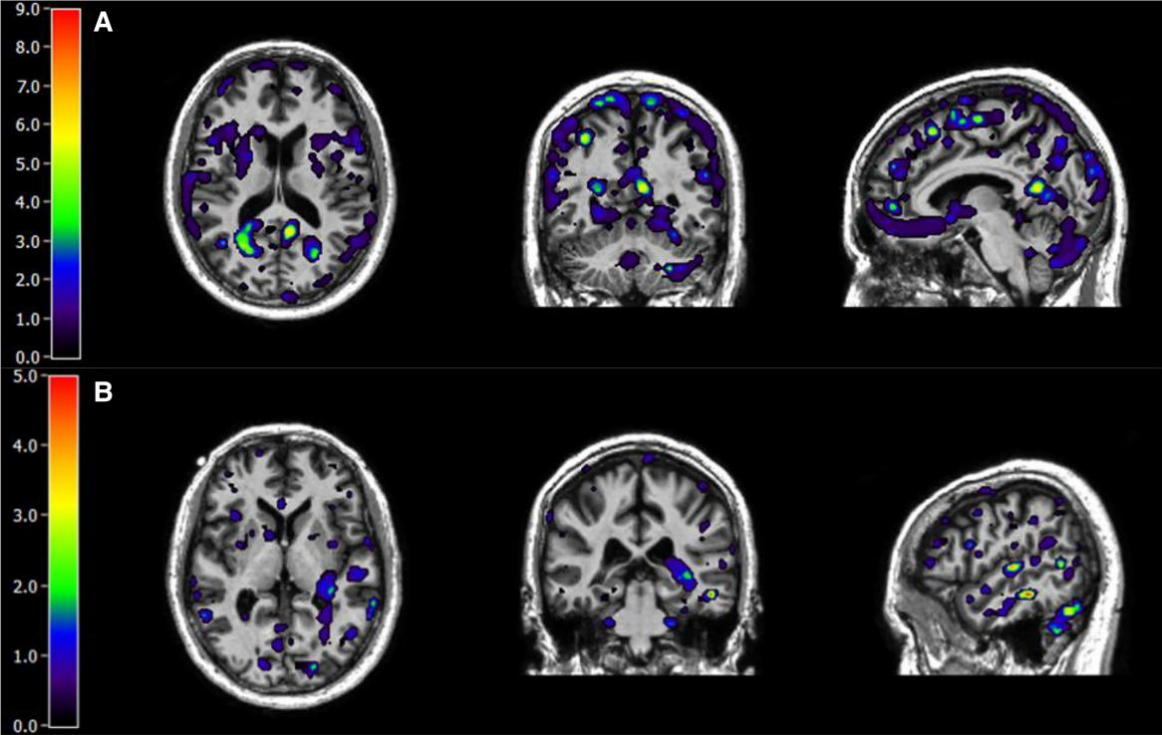
**Variability in the individual patterns of hypometabolism (A) and hypermetabolism (B) in TGA patients.** The colour bar reflects the frequency (from 0 = absent in all patients to 10 = present in all TGA patients) of significant hypo- or hypermetabolism in each voxel of the patients’ brains. The scale reaches nine for hypometabolism and five for hypermetabolism. No voxels are found to be altered in all patients.

Regarding hypometabolism, there was no brain region systematically affected in all TGA patients (see individual *Z-Score maps* in Fig. 1 and *Z-*Scor*e frequency maps* in Fig. 2A and B). Hypometabolic similar clusters of a significant size (minimum of 25 voxels) were found in only three patients in the right middle frontal gyrus. The pattern of brain hypermetabolism was even less consistent with each cluster of a significant size found only in one patient at a time.

### Correlation between brain metabolism and temporal aspect

In TGA patients, there was no significant correlation between glucose metabolism uptake and the calculated ratio at *P* < 0.001 (uncorrected for multiple comparisons).

### Correlations matrix

**Figure 3:**
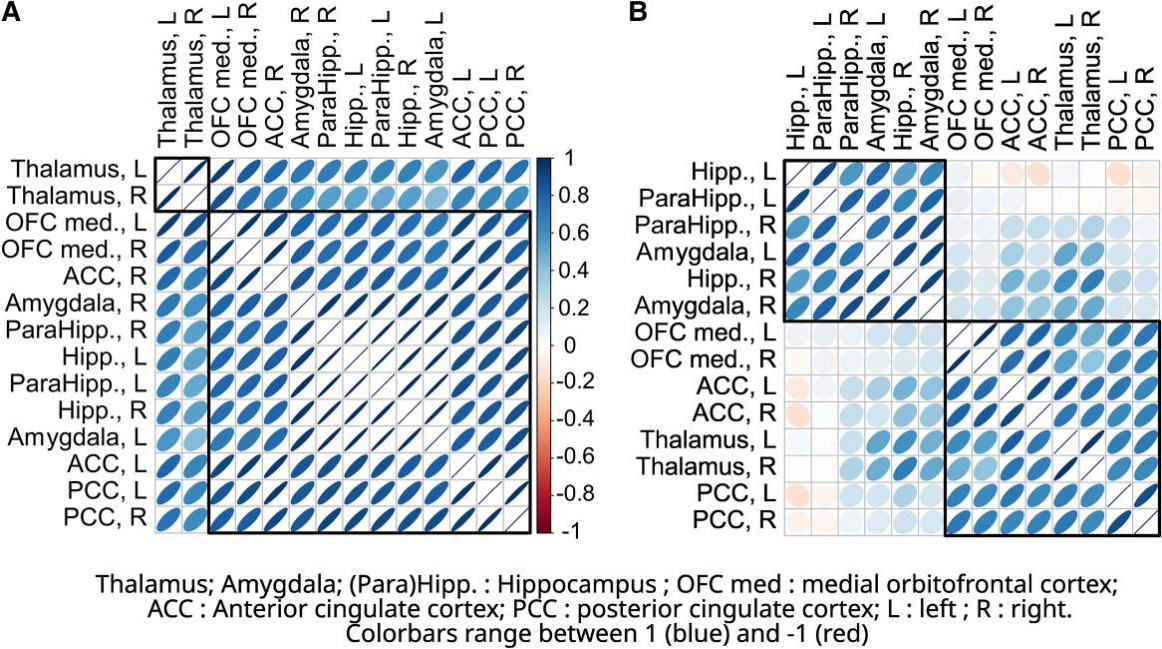
**Correlations matrix of the regional metabolism in controls (A) and TGA patients (B).** Positive correlations are shown in blue and negative correlations in red. The narrower the ellipse, the closer the *r_BP_* value is to 1 or −1. The darker the ellipse, the stronger the correlation between the pair of metabolic measurements. The two main clusters are marked in black. L = left, R = right. ACC = anterior cingulate cortex; Hipp = hippocampus; OFC med = medial orbitofrontal cortex; ParaHipp = parahippocampal gyrus; PCC = posterior cingulate cortex. Colourbars range between 1 (blue) and −1 (red).

**Figure 4:**
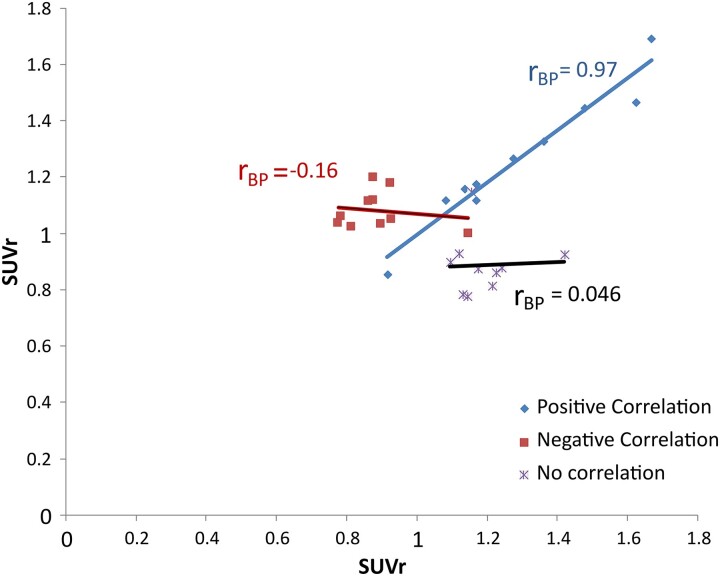
**Interpretation of ellipses in correlational matrices of** Fig. 3. Three examples are shown. First, in controls, the high and positive correlation between the left and right thalami (*r_BP_* = 0.97), depicted in blue here, is represented by a narrow and dark blue ellipse in Fig. 3A. In the second example, the correlation observed in patients between the left hippocampus and right anterior cingulate gyrus, depicted in red here, is negative and weak (*r_BP_* = −0.16). It is represented in Fig. 3B by an ellipse that takes the shape of a circle and a light shade of red. In the third example, the correlation depicted in black here and observed in patients between the left thalamus and left hippocampus is close to zero (*r_BP_* = 0.046) and represented in Fig. 3B as a white square. Axes denote standardized uptake value ratios (SUVr).

The correlation matrices (Fig. 3A and B) are depicted as a grid of ellipses. Positive correlations are depicted in blue and negative ones are in red. The shade and width of the ellipses illustrate the strength of the correlation for each pair of regions studied. As the correlation coefficient, $r_{BP}\underset{}{\to }-1$, the ellipse becomes narrower and takes a darker shade of blue. For example, as further illustrated in Fig. 4, from Fig. 3A, the left and right thalamus correlate highly and positively with each other (*r_BP_* = 0.97) and the ellipse is narrow and dark blue. Similarly, when $r_{BP}\underset{}{\to }-1$, again the ellipse becomes narrower as the scatter condenses but takes a darker shade of red (for example, from Fig. 3B, where *r_BP_* = −0.16 between left hippocampus and right anterior cingulate gyrus). As $r_{BP}\underset{}{\to }\;0$, the scatter plot becomes more sparse and assumes a lighter shade until it ultimately shows as a white square, hence indicating no significant correlation (for example, from Fig. 3B, *r_BP_* = 0.046 between left thalamus and left hippocampus). Examples of *r_BP_* are shown in Fig. 4. All statistical analyses have been corrected for multiple comparisons using Bonferroni (all associated *P*-values were multiplied by 14 and results were considered to be statistically significant when *P* < 0.05).

The hierarchical clustering method enabled the identification of two clusters of highly intercorrelated brain metabolic regions.

In controls (Fig. 3A), even though the analysis showed two clusters, all regions included in the matrix highly and positively correlated with each other. For example, metabolism of the left thalamus (cluster 1) significantly and positively correlated with metabolism in the left OFC (cluster 2, *r_BP_* = 0.86; *P* < 0.001).

In the patient group, the pattern was different (Fig. 3B) with a clear dissociation between the two highlighted clusters. Regions of the medial temporal lobe (the hippocampus, parahippocampal gyrus and amygdala) were included in the same cluster (cluster 1). The OFC, anterior and posterior cingulate gyrus and thalamus were gathered in the other cluster (cluster 2). Remarkably, those two clusters were weakly correlated to each other, sometimes negatively, with the absence of significant correlation between regions of cluster 1 with most of the regions of cluster 2. For example, metabolism in the left hippocampus (cluster 1) did not correlate to metabolism in the left posterior cingular gyrus (cluster 2, *r_BP_* = −0.09, *P* = 0.666).

## Discussion

The brevity of TGA makes it very difficult to study its pathophysiological mechanisms at an early stage, which explains the scarcity of neuroimaging studies conducted in these patients and the fact that they are limited either to case studies or to group studies where patients have been studied after the acute phase. In that context, our study provides novel data that has been scrupulously acquired over the past 5 years, all under the same conditions and having the same clinical, neuropsychological and neuroimaging examinations. Using this rare dataset of FDG-PET data collected in a group of ten TGA patients (three in acute and seven in recovery phase), the present study aimed at investigating the contribution of glucose metabolism measure to the study of pathophysiological mechanisms underlying amnesia and anxiety in TGA.

Contrary to previous case studies having used an ROI approach and having reported regional hyper or hypometabolism,^11-15,40^ the present voxel by voxel investigation of glucose metabolism in the whole brain did not reveal any significant differences between TGA patients and healthy controls. This result could first be explained by the heterogeneity of the TGA sample. Indeed, even though TGA symptoms are usually stereotypically described, patients differ regarding the total length of the acute phase, the rapidity of the recovery phase, the arrival time at the emergency department, or the delay between the PET scan and the TGA onset. The analysis of variability within the TGA group confirms the absence of consistency regarding the pattern of metabolic abnormalities with no region being systematically significantly affected in all patients (Fig. 2). We investigated the potential role of some factors such as the earliness of the PET scan. For example, we examined the relationships between regional glucose metabolism of TGA patients and a ratio considering the delay between the supposed onset of amnesia and the PET scan. The absence of significant correlations does not provide any further explanation regarding between-subject variability. Visual examination of TGA individual PET results (Z-scores maps, Fig. 1) confirms that the patients who underwent the PET scan during the acute phase did not exhibit more severe metabolic abnormalities than those examined at the end of their episode. Examining a transient amnesia, which by definition does not lead to severe long-term deficits, requires the capture of very short-lived phenomena. DWI studies highlight the difficulty of detecting brain changes in the acute phase. Focal hippocampal lesions found in TGA patients in the CA developed between 24 and 48 hours following the onset of TGA. However, in a recent meta-analysis on DWI data, results showed that in a substantial number of patients with TGA, there were no visible DWI lesions of hippocampus even though they were amnesic.^41^ These data suggest that TGA is associated with functional deficits of the brain that are more threshold-dependent^4^ than with the time (acute or post-acute) of the amnesic phase. A recent study using resting-state functional MRI in TGA showed altered functional connectivity, which can be observed when patients are compared to a control group, but cannot be detected when patients are compared according to the time (acute or post-acute) of the TGA phase.^42^ Given the individual variability in the time course of TGA, the direct comparison between a group of patients and controls does not seem to favour the identification of subtle and transient alterations in regional metabolism.

The absence of a clear pattern of metabolic alterations may also potentially be explained by the specific nature of the pathophysiological mechanisms involved in TGA. DWI studies suggest that functional and reversible alterations of the hippocampus may play a key role even though it may not be directly detected by a lower or higher glucose uptake in that region. A transient dysfunction of cortical spreading depression triggered by excessive glutamate release within an extended hippocampal network has also been hypothesized.^43^ Indeed, CA1 neurons are known to be vulnerable to metabolic and oxidative stress that may be caused by an overload of cellular glutamate.^44^ This would lead to a neural dysfunction with a particular mode of propagation within the hippocampus and more largely through the limbic circuit, potentially resulting in TGA. The above arguments, therefore, potentially favour the theory of metabolic dysfunction at a network level instead of being centred over a particular region. Indeed, a growing number of studies^30,45^ indicates not only the involvement of the hippocampus in episodic memory but also that of an extended network, suggesting that memory impairments could be considered as resulting from reduced network connectivity (for review see Jeong *et al*.^46^) or altered coordinated action within that network.^47^ The involvement of an extended network in the pathophysiology of TGA would also explain the other symptoms observed, such as mood changes and notably anxiety which is frequently observed in association with amnesia during the acute phase. Moreover, from a methodological point of view, using correlations instead of direct comparisons can help us to better understand networks mechanisms. Indeed, metabolism correlations between regions will stay supported regardless of the temporality of the TGA phase in which the patient is. In accordance with this assumption, our findings indicate differences in the functional connectivity between brain regions within the limbic circuit^28,29^ in controls versus TGA patients. In healthy controls, regions of the limbic circuit seem to operate in a synchronized way with all regions being highly correlated to each other. On the opposite, in TGA patients, we observed a clear disruption of the correlational patterns between the two clusters, with the hippocampus, parahippocampal gyrus and amygdala being classified in one cluster that poorly correlated with the other brain regions of the limbic circuit. In terms of brain networks, such a disruption in the correlational patterns between the amygdala and the rest of the limbic regions could explain the frequency of anxiety in TGA patients. Interestingly, anxiety frequently observed during the TGA episode, decreased, conjointly with the level of salivary cortisol,^18^ in parallel with episodic memory recovery.^2^ These observations support the idea that memory and emotional changes may be related. Such an alteration in synchronization within and between regions of the limbic system shows its dysfunction at a network level and could explain the association of memory disorders and anxiety observed in these patients during the acute phase.

In agreement with our hypothesis, previous studies also reported changes in brain networks in TGA even though they did not focus on the limbic system. In 12 TGA patients seen in the acute phase, Peer *et al*.^25^ reported a reversible reduction in functional connectivity in data-driven and literature-based episodic memory networks. This altered functional connectivity was more pronounced during the acute phase compared to the recovery phase and was not present anymore after recovery. Using single photon emission computed tomography, hypoperfusion was found in the left precuneus and inferior temporal and superior parietal areas in a group of 22 TGA patients.^48^ The authors concluded that these results may be related to a disruption of the posterior medial network,^49^ also described as involved in episodic memory.^50^ Another study,^24^ using FDG-PET in TGA patients, 24–72 hours after onset, also reported decreased metabolism in the posterior medial network, suggesting that this network alteration may explain the symptoms appearing earlier, during the acute phase. Park *et al*.^22^ investigated brain networks using diffusion tensor imaging and found altered structural connectivity and reorganization of network hubs in TGA patients, especially in the regions of the default-mode network.

Taken together, these studies and ours support the idea of a network disruption playing a key role in the pathophysiology of TGA. This study deepens our understanding of the mechanisms underlying not only amnesia but also the emotional component of TGA by considering it as a disruption in the correlational patterns within the limbic circuit.

### Technical and methodological considerations

The sample size and the heterogeneity of the TGA group are the main limitations of the present study. However, TGA is a sudden and transient syndrome that makes difficult the recruitment of patients, especially in a PET study. As far as we know, our study is the first to investigate metabolic networks within the limbic system in a group of ten patients examined during their TGA episode.

## Conclusions

To conclude, our findings indicate that the synchronization of regions within the limbic circuit is altered during the acute phase of TGA. A transient disruption of the correlational patterns between those regions is consistent with the reversible nature of amnesia and the frequently associated anxiety observed in TGA patients. Our study thus offers a new approach to investigate the pathophysiological mechanisms underlying TGA and suggests that memory disorders and anxiety may be two expressions of the same transient network dysfunction.

## Supplementary Material

fcad082_Supplementary_Data

## Data Availability

Data are available from the corresponding author, upon reasonable request. Data are not publicly available due to ethical restrictions because the information contained on those data could compromise the privacy of the reported patients.

## Abbreviations

AAL =: automated anatomical labelling
CA =: cornu ammonis
CHU =: Caen University hospital
CMRO2/glu =: cerebral metabolic rates for oxygen/glucose
DWI =: diffusion-weighted imaging
ESR =: encoding – storage – retrieval
FDG-PET =: [18F]-2-fluoro-2-deoxy-d-glucose
MNI =: Montreal neurological institute
NA =: not available
OFC =: orbitofrontal cortex
ROI =: region of interest
SPM =: statistical parametric mapping
STAI =: state-trait anxiety inventory
TGA =: transient global amnesia
VBM =: voxel-based morphometry
WMS =: Wechsler’s memory scale
