# Supplementary material for: Disruption in normal correlational patterns of metabolic networks in the limbic circuit during transient global amnesia
Source: Brain Commun. 2023 Mar 21;5(2):fcad082. doi: 10.1093/braincomms/fcad082 (PMC10123398; doi:10.1093/braincomms/fcad082)
Supplement: fcad082_Supplementary_Data [file fcad082_supplementary_data.pdf]

Supplementary data

Supplementary data 1. Detailed TGA onset individual context.

PA01

Patient 01 was a 72 year-old right-handed woman, retired psychologist. The medical record reports that she was usually an anxious person. The patient came to the hospital for a 6 months follow-up control of a stomach surgery that did not show any problems. This medical visit did not come with any specific emotional reaction. The TGA episode started at one p.m. when PA01 left the appointment and her husband noticed iterative questions. PA01 arrived at the emergency department at 1:32 p.m. with her husband and the neuropsychological examination began around 2:40 p.m. The clinical interview showed a massive anterograde amnesia, a retrograde amnesia that covered at least the past three days (she did not remember a snow problem) and iterative questions about place and the current situation ("Where am I?", "Who brought me here?", "Where is my husband?", "What is the matter with me?"). The acute episode was accompanied with a sensation of cold feet. The patient was no aware of her memory problem. PA01 started to recover around 8 p.m. The PET scan was carried out at 4:30 p.m., when the patient was still in the acute phase.

PA02

Patient 02 was a 59 year-old right-handed woman, who worked as a work inspector. She had no previous medical history except for a phlebitis 15 years earlier. She was getting ready as every day and just finished to drink a coffee when the episode started at 8:30 a.m. without any particular precipitating event. Her relatives noticed that she started asking the same questions repeatedly. The neuropsychological examination started at 11 a.m. It showed a retrograde

amnesia over a few days ago, but the patient remembered her job change 3 weeks ago (she had been previously working as a health and safety inspector for twenty years). She was not aware of her amnesia, and she admitted she had difficulties only after failing at the WMS test. The TGA episode was accompanied with iterative questions about the date and place and it lasted until 4:30 p.m. The PET scan was carried out at 1:30 p.m., when the patient was still in the acute phase.

PA03

Patient 03 was a 65 year-old right-handed woman, who worked as a nurse/child-minder. She had a history of arterial hypertension and migraine and had an episode of depression 20 years ago. According to her medical record she was generally anxious. The day of the TGA, the patient had an argument with her ex-husband on the phone earlier in the morning (10 a.m.). They divorced had 3 months ago and the call was very stressful and intense. The patient was getting ready and finished to dress up when the episode started, at 10:30 a.m. She just saw her neighbor who noticed nothing unusual and she called her daughter. On the phone, she declared that something was wrong without specifying a memory problem and started to ask iterative questions. Her daughter took her to the hospital for 11:51 a.m. where the neuropsychological examination started at 2:10 p.m. The episode was accompanied with iterative questions about place and worries (“How did I get here?”, “Does someone take care of the children?”). PA03 realized having a memory problem only after failing at the story recall test. The episode ended at 3:30 p.m and the PET scan was done at 4:15 p.m.

PA04

Patient 04 was a 69 year-old right-handed retired woman, with anxiety according to her medical record. The acute phase started at 9 a.m. after taking a shower. She called her daughter, speaking

of a general unease and her daughter immediately noticed a memory problem and iterative questions (“I don’t know if I’m waking up of a dream”, “I’m going out of the bathroom”). The patient arrived at the hospital at 11:54 a.m. and the neuropsychological examination started at 1:30 p.m. An important retrograde amnesia was noticed, the patient could not remember the last Christmas, 8 months ago. PA04 asked iterative questions about place and her current situation (“Where am I?”, “Did I take these clothes?”). The patient spontaneously talked about her memory problems. The recovery started around 6 p.m. and the PET scan was done at 8 a.m. the next morning. Interviewed after the TGA about the context of the amnesia’s onset, the patient reported a dispute between her daughter and grandchildren one week ago.

PA05

Patient 05 was a 61 year-old right-handed woman, retired nurse. She had a history of thyroid disease and she was naturally anxious according to her medical record. The TGA episode started at noon when she was at the swimming pool. At the end of the session, when she was going to take a shower, she cannot remember where she put her clothes. At 12:40 p.m., she arrived at her house and her husband noticed she didn’t recognized the house or her daughter’s boyfriend. The patient arrived at the hospital at 1:46 p.m. and the neuropsychological examination started at 2:50 p.m. The patient reported a headache during the attack. She could evoke a memory problem but only if she was specifically asked about it. The episode lasted until 7 p.m. The patient underwent the PET scan at 4:30 p.m. After recovery, she reported that she had been worried for a month about the health of her mother, who had entered the hospital the day before TGA. Two months later, when she was skiing, PA05 had another TGA episode that lasted 4 hour (from noon to 4 p.m.).

PA06

Patient 06 was a 60 year-old right-handed woman, who had been retired for 5 months. She had a head trauma after a motorcycle accident, a loss of consciousness 12 years before after a strangulation and a medically treated anxiety disorder for a month, 25 years ago. On the day of the episode, PA06 made coffee at 9 a.m. and she took a shower at 9:30 a.m. At 9:45 a.m., she told her daughter "I'm feeling weird". The daughter noticed iterative questions ("What day is it?", "What are we doing this weekend?"). At 10:57 a.m., they arrived at the hospital and the neuropsychological examination started at 12:23 p.m. The patient had no memory of her daughter's birthday 4 days ago. During the examination, the patient complained of headache, and showed high emotionalism and with episodic crying. She asked repetitively about the date and worries ("What day is it?", "What time is it?", "What are we doing this weekend?"). The episode lasted until 1 p.m. She underwent the PET around 4:40 p.m. After having recovered, the patient reported that in the past three days, her son was at the hospital because of having got drunk at a party.

PA07

Patient 07 was a 69 year-old right-handed retired woman. She had a history of arterial hypertension and had migraines until the age of 53. On the 31stof December, she went to a New Year's Eve dinner where everything went fine. At 5:30 a.m. she answered a phone call from her brother. In the morning when she woke up, around 10 a.m., her husband noticed something wrong. The patient could not remember the past day. PA06 arrived at the hospital at 12:04 p.m. and the neuropsychological examination started at 2:30 p.m. The patient asked iterative questions and repetitively said "I have a black hole". She was aware of her memory problems. The episode lasted until 1 p.m. The patient kept no memory of the New Year's Eve dinner even after having recovered from her amnesia. The PET scan was done at 1 p.m. the following day.

PA08

Patient 08 was a 54 year-old right-handed woman with no previous medical history. The patient woke up at 6 a.m., had breakfast, took a shower and left to work at 7:10 a.m. She arrived at work at 7:30 but did not feel well. At 8:15 she realized she could not remember her colleagues' name. She continued her day at work but, in the hallways, she felt like a suffocative feeling, that she qualified as a "claustrophobia crisis". She phoned her husband at 9:30 a.m. to tell him she could not remember her colleagues' name. Her colleagues took her in an office and she had a loss of contact for two minutes. Firefighters arrived at 10:30 and drove her to the hospital. The panic attack could have been a precipitant factor of the TGA episode. The neuropsychological testing started at 12 p.m. She could not remember her present address where she had moved 3 weeks ago. After having recovered, the patient remembered the firefighters and spoke in the ambulance but she had a lacunar amnesia from the time of her arrival at the emergency department until the neuropsychological tests. There were no iterative questions but she worried about forgetting the names and her new address. PA08 seemed perplexed, lost in thoughts, with problems of concentration and difficulties to focus. The episode lasted until 2 p.m. The PET scan was done at 2:15 p.m.

PA09

Patient 09 was a 67 year-old left-handed retired man. He had a cranial trauma 17 years before and had been treated since for dizziness. His wife left the house at 9:45 a.m. A friend called him right after and found that PA07 was not feeling well. At 10 a.m., another friend came to visit him. The patient recognized him but started to ask iterative questions ("What day is it?", ten times, "Why are you here?"). No precipitating event was noticed except for dizziness the day before. The patient arrived at the hospital at 12:13 p.m. and the neuropsychological

examination started at 1:15 p.m. The retrograde amnesia extended over one week. PA09 asked iterative questions about the date and his current situation. The episode lasted until 6 p.m. The patient underwent the PET scan at 9 a.m. the next morning.

PA10

Patient 10 was a 62 year-old right-handed man who had been retired for 2 years. He had a history of anxiety disorder at the age of 30 and a depression episode 25 years before the TGA. He had been diagnosed with cancer four years before the TGA episode. He had spent some time in an intensive care unit, received morphine and was put in a coma during three weeks. Since that time he had trouble dating events. PA10 woke up at 7 a.m., had breakfast and went back to bed. The TGA episode started approximately at 10 a.m. At that time, his daughter called her mother to report that PA10 was unable to tell the date of the day. The patient arrived at the hospital at 10:45 a.m. and the neuropsychological examination started at 3 p.m. The patient remembered nothing of the week before. During the episode, the patient had nausea and a highly emotional state with cries. He asked repetitively about the date. The PET scan was done at 9 a.m. the next morning. After having recovered, the patient reported that he had conflicts with his daughter two weeks ago and that, the day of the episode, he was waiting for his daughter’s school exams.
